# A Rare Case of Staphylococcus carnosus Bacteremia and Discitis

**DOI:** 10.7759/cureus.112570

**Published:** 2026-07-13

**Authors:** Suhel A Sabunwala, Tuhina Cornelius

**Affiliations:** 1 Infectious Diseases and Global Medicine, University of Florida, Gainesville, USA

**Keywords:** coagulase- negative staphylococcus, lower back pain (lbp), staphylococcus species, vertebral discitis, vertebral-osteomyelitis

## Abstract

Spinal discitis osteomyelitis is commonly caused by hematogenous spread of *Staphylococcus aureus* to the disc space. Coagulase-negative Staphylococcus species, particularly *Staphylococcus carnosus* has not been reported as the cause before.

We report the case of an 84-year-old male patient with a medical history of hypertension and coronary artery disease who presented with a complaint of lower back pain for two weeks before presentation. MRI of the lumbar spine showed findings of marrow edema and T1 hypointensity of the L3 -4 vertebral bodies, raising suspicion for discitis and osteomyelitis. An extensive work-up for infection was initially negative, and the patient was treated with broad-spectrum IV antibiotics for six weeks; however, two months after completion of the IV antibiotics therapy, his symptoms recurred. He then presented back to the hospital with worsening lower back pain and weakness and was found to have *Staphylococcus carnosus* bacteremia. The patient was treated with IV daptomycin and IV cefazolin, followed by oral doxycycline, with improvement in his symptoms.

To our knowledge, this is the first reported case of* Staphylococcus carnosus* bacteremia resulting in discitis and osteomyelitis. This case highlights the importance of keeping coagulase-negative Staphylococcus species, such as *Staphylococcus carnosus*, as causative pathogens for discitis osteomyelitis, even in immunocompetent patients. This case also underscores the need for a prolonged course of antibiotics to prevent the recurrence of infection and related morbidity and complications.

## Introduction

Vertebral discitis osteomyelitis is defined as infection of vertebral bodies and disc space in the spine. There could also be involvement of facet joints. This most commonly arises from hematogenous spread; however, it can also occur after trauma resulting in open wounds and chronic pressure-related decubitus wounds in the elderly population with dementia or patients with neurological impairment. Use of IV drugs, endocarditis with valve vegetations, immunosuppression status, uncontrolled diabetes mellitus, and active malignancy are some of the risk factors for hematogenous spread of infection to the spinal disc space and vertebrae [1]. Vertebral discitis osteomyelitis is encountered more commonly in clinical practice than before due to an increase in spinal surgical interventions, increased percutaneous interventions, and intravascular procedures [2]. *Staphylococcus aureus* is the most common causative pathogen and usually results from the hematogenous spread of the infection. MRI is the imaging modality of choice for the diagnosis and evaluation of the extent of bone and soft-tissue involvement. In particular, MRI has a high sensitivity and specificity for pyogenic discitis [3]. Blood cultures are positive in 20-60% of the cases [4]. CT-guided biopsy and cultures can identify the causative pathogen in approximately 70-90% of cases [5]. The duration of antibiotic therapy ranges from 4-12 weeks [6].

In this manuscript, we report a case of coagulase-negative Staphylococcus species - *Staphylococcus carnosus -* as the causative organism for discitis osteomyelitis of L3-4 vertebrae. Despite treatment with six weeks of IV antibiotics, there was a relapse of infection requiring hospitalization and a repeat prolonged course of IV antibiotics followed by oral antibiotics.

## Case presentation

**Figure 1:**
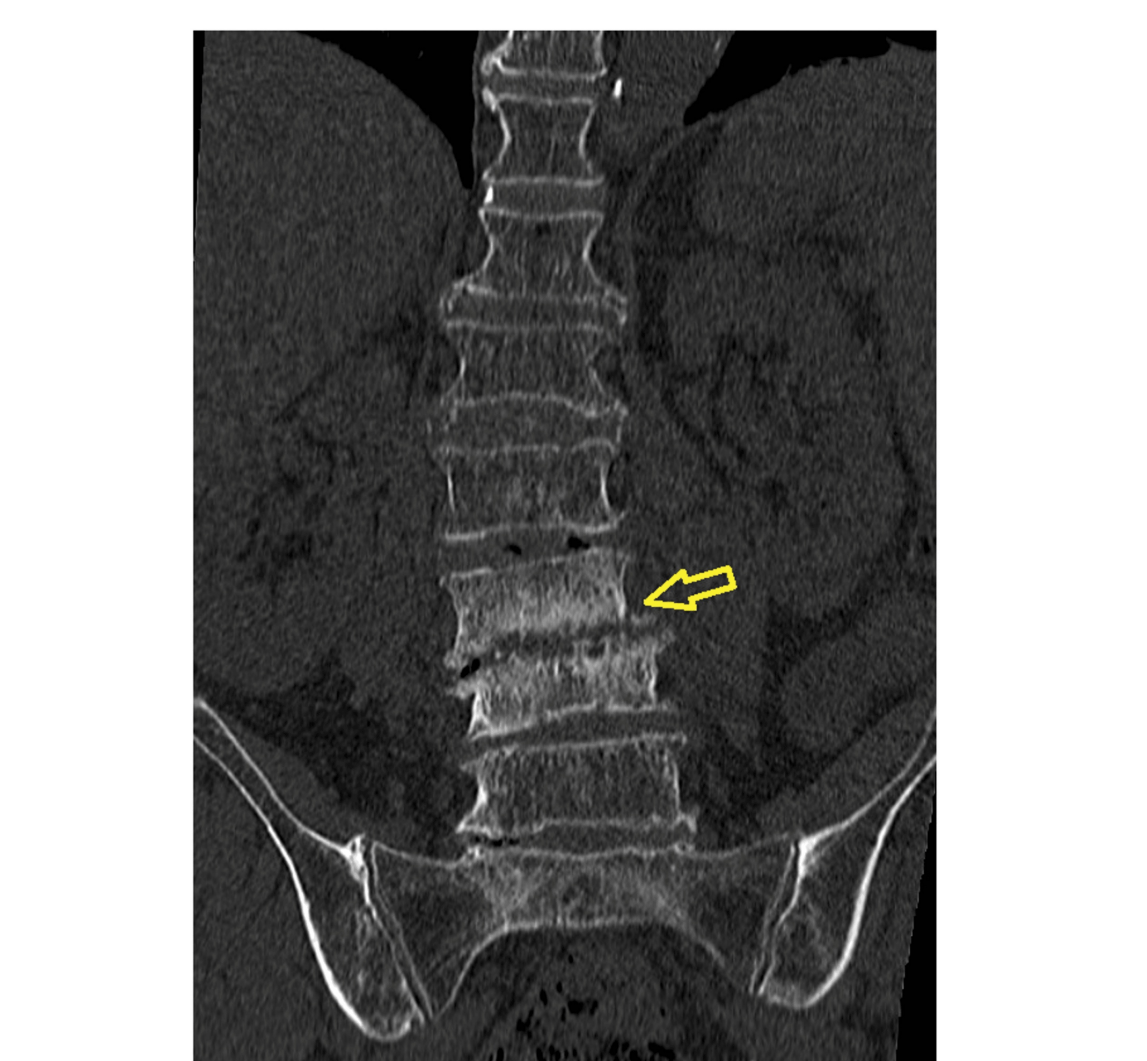
CT of the lumbar spine, coronal view Arrow highlights L4 anterior superior endplate compression fracture with height loss and associated non displaced fractures of the L4 inferior articular processes. Additional findings present for advanced multilevel spondylosis.

An 84-year-old male patient with a history of hypertension, coronary artery disease requiring percutaneous coronary interventions, and depression presented with a complaint of two weeks of lower back pain radiating to his legs with associated bilateral hip and anterior thigh pain. His symptoms initially began shortly after lifting a 5-gallon water bucket. The patient denied any other complaints, including fever, chills, trauma, or falls. He denied any prior history of bloodstream infection or any prior back surgery. Upon presentation to the emergency room (ER), he was afebrile. Initial laboratory results were unremarkable except for a slightly elevated erythrocyte sedimentation rate (ESR) and C-reactive protein (CRP) (Table 1). CT of the lumbar spine revealed an acute to subacute L4 anterior superior endplate compression fracture and advanced multilevel spondylosis with severe spinal canal stenosis (L3-4 and L4-5) (Figure 1).

**Table 1:** Summary of the pertinent lab findings from all the hospital visits ^1^Tissue specimen was sent for bacterial, fungal, and AFB cultures; ^2^Tissue specimen was sent for bacterial, fungal and AFB NGS PCR testing WBC: White blood cell; CRP: C-reactive protein; ESR: Erythrocyte sedimentation rate; NGS: Next-generation sequencing; PCR:  Polymerase chain reaction; AFB: Acid-fast bacilli

Labs	Reference Range	Hospitalization 1 (5/25)	Hospitalization 2 (7/25)	Hospitalization 3 (12/25)
WBC	4.0-10.0 x10^3^/µL	7.5 x10^3^/µL	11.8 x10^3^/µL	15.4 x10^3^/µL
CRP	0.20-5.00 mg/L	12.11 mg/L	13.48 mg/L	125.88 mg/L
ESR	0-10 mm/hour	40 mm/hour	22 mm/hour	42 mm/hour
Blood Cultures	No growth	No growth	Staphylococcus hominis	Staphylococcus carnosus
Tissue Cultures^1^	No growth	No growth	No growth	Not Analyzed
Tissue NGS PCR^2 ^	Negative	Negative	Negative	Not Analyzed
Histoplasma Antigen/Antibody	Negative	Not Analyzed	Negative	Not Analyzed
Brucella Antibody	Negative	Not Analyzed	Negative	Not Analyzed

**Figure 2:**
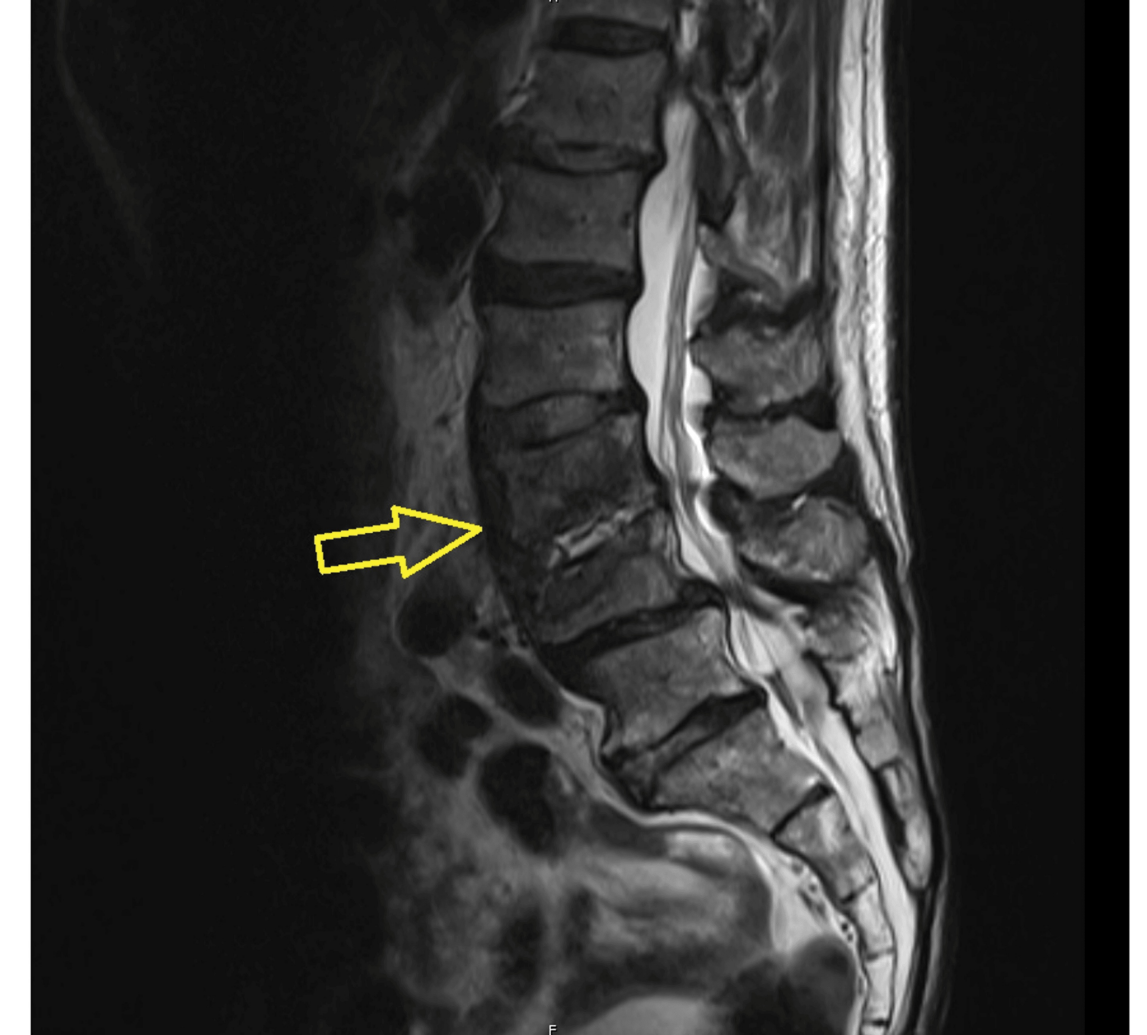
Sagittal T2-weighted MRI of the lumbar spine Arrow highlights diffuse enhancement of L3 and L4 which extends into the pedicles. There is also edema in the pre vertebral soft tissues at L3 and L4. Additional findings on the MRI include multilevel degenerative changes and ligamentum flavum hypertrophy present.

The neurosurgery team evaluated the patient and recommended continuing supportive care with a lumbar brace and pain control. His symptoms did not improve, and an MRI of the lumbar spine was performed, which showed marrow edema and T1 hypo intensity of L3-4 vertebral bodies and diffuse enhancement of L3-4; also noted were edema and enhancement of a possible infiltrating process in the prevertebral soft tissues at L3-4 with an enhancing ventral epidural soft tissue lesion with possible compression. There was no fluid enhancement in the L3-4 space, making discitis unlikely (Figure 2).

A CT-guided biopsy was performed of the L3-4 disc space, and specimens were sent for bacterial, fungal, and acid-fast bacilli (AFB) cultures along with molecular polymerase chain reaction (PCR) testing for 16s rRNA next-generation sequencing (NGS) test at the University of Washington Molecular Lab. All cultures and PCR tests were negative, and a decision was made to monitor off any antibiotics and to continue supportive care with pain management.

**Figure 3:**
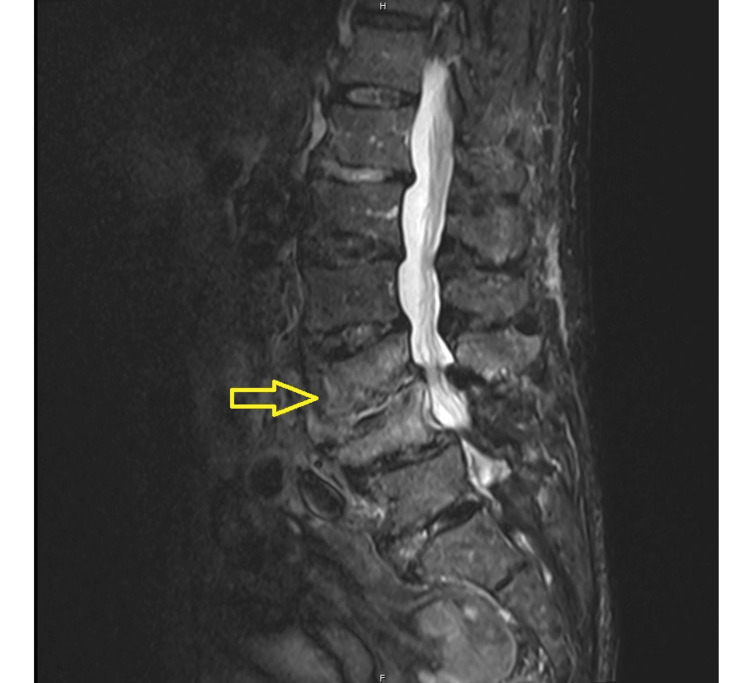
Sagittal contrast-enhanced MRI of the lumbar spine Arrow highlights diffuse marrow edema in the L3 and L4 vertebral bodies with decreased intervertebral disc space. There is enhancing ventral epidural tissue at L3-4 without discrete non enhancing collection. These findings are suggestive of discitis osteomyelitis without discrete abscess.

The patient then presented with similar complaints approximately six weeks after the initial presentation. MRI of the lumbar spine was repeated and showed diffuse marrow edema at L3 and L4 with some degree of enhancement in the prevertebral and ventral epidural space, suggesting ongoing discitis osteomyelitis without a discrete abscess (Figure 3).

**Figure 4:**
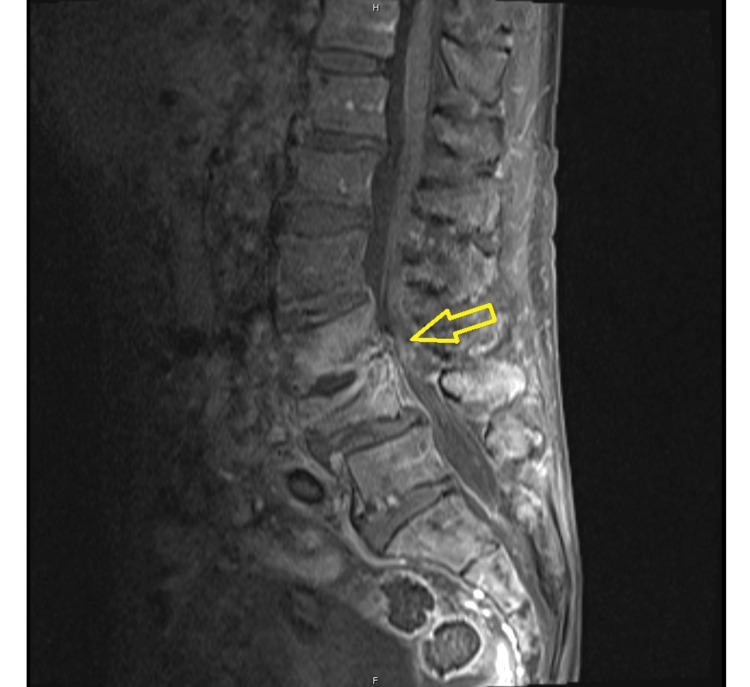
MRI of the lumbar spine, sagittal view Arrow highlights collapse of superior endplate of the L4 vertebra. There is prominent enhancement of the vertebral bodies with enhancing paraspinal granulation tissue, as marked with the arrow head.

Patient had similar infectious workup as the previous hospitalization that resulted negative; however, two sets of blood cultures sent from separate sites on this hospital visit returned with *Staphylococcus hominis *in two out of four bottles (both aerobic) (Table 1). The patient was discharged with six weeks of intravenous daptomycin (8mg/kg) and IV Ceftriaxone (2 g daily). He was followed up after five weeks in the outpatient clinic with minimal improvement in back pain; however, CRP levels normalized, and antibiotics were discontinued after the planned six-week course. MRI of the lumbar spine was repeated at the end of IV antibiotics therapy, and again after two months, which showed evolving changes of discitis and osteomyelitis at the L3-4 level with collapse of endplates and persistent unchanged paravertebral enhancing granulation tissue (Figure 4).

The patient presented after four months with complaints of feeling unwell and generalized weakness with malaise. He was diagnosed with sepsis. Blood cultures returned gram-positive cocci in four out of four bottles and were later identified as *Staphylococcus carnosus*. Lab results from the hospital visits are summarized in Table 1.

MRI of the lumbar spine showed evolving changes of L3-4 spondylodiscitis with suggestion of mildly increased paravertebral soft tissue, ventral epidural space edema, and inflammation compared to the previous MRI scans (Figure 2).

**Figure 5:**
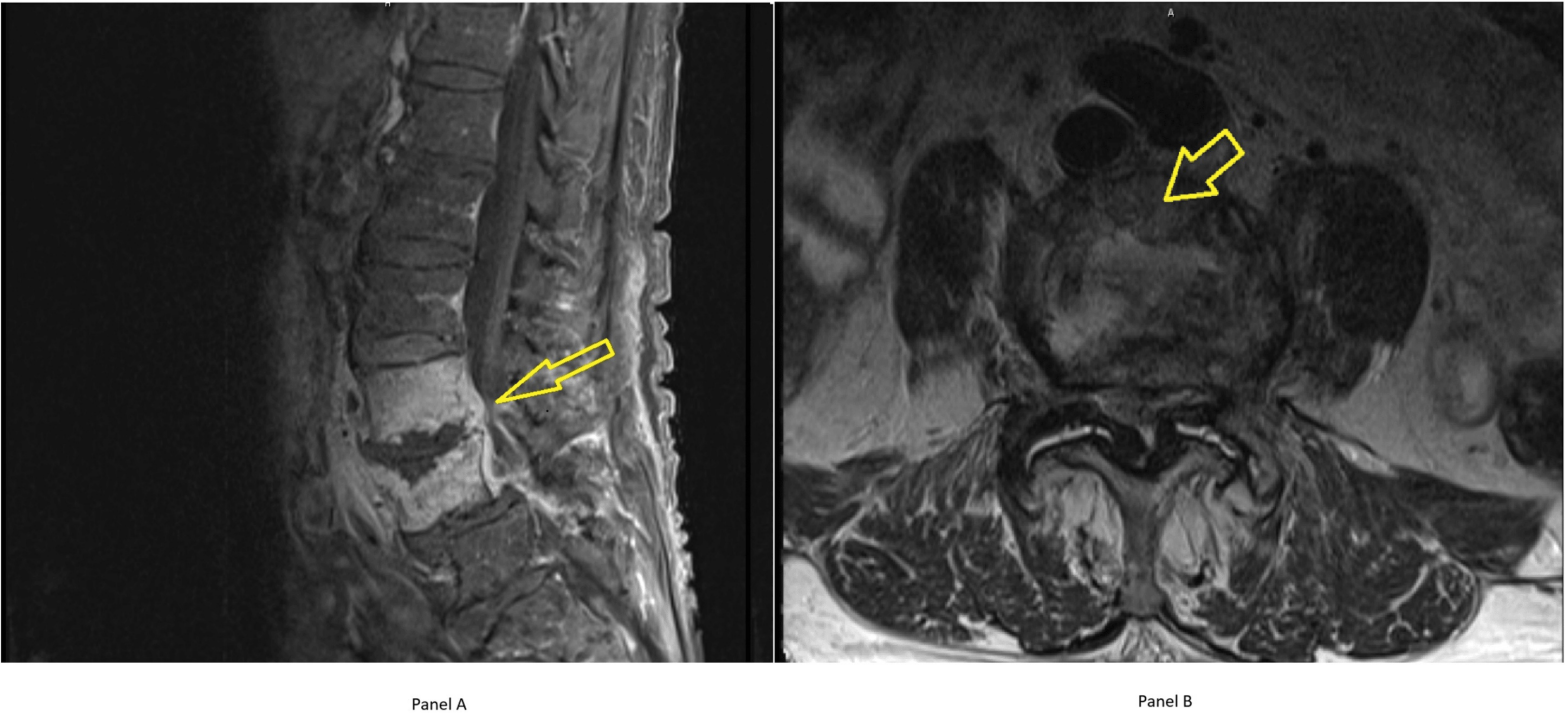
MRI of the lumbar spine Panel A is the sagittal section, in which the arrow highlights marked prevertebral and paravertebral soft tissues edema and enhancement, the degree of which is increased compared to the previous scan; Panel B is the transverse section, in which the arrow highlights destructive process centered at the L3-4 intervertebral disc space with vertebral endplate erosions. These findings have worsened when compared to the previous MRI.

Transesophageal echocardiogram was negative for valve vegetations or any other signs of endocarditis. Blood cultures after 24 hours of culture-directed antibiotic treatment were positive; however, repeat blood cultures after 48 hours and 96 hours from the initial cultures were negative. The patient was discharged with six weeks of IV daptomycin (8 mg/kg) and IV cefazolin (6 g of continuous infusion daily). After completion of the course of IV antibiotics, the patient was started on oral doxycycline 100 mg twice a day, which was well tolerated at three months follow up visit. The patient showed improvement in lower back pain; however, the pain did not resolve completely. Blood cultures obtained showed no growth during oral doxycycline therapy. He continues to follow up with the neurosurgery team for pain management and ongoing discussions on surgical interventions for compression fracture.

## Discussion

*Staphylococcus carnosus* is generally considered a nonpathogenic Staphylococcus species. *Staphylococcus carnosus* is used as a starter culture in the food industry and is recognized as a generally non-pathogenic organism [7]. There is evidence of various genes in *Staphylococcus carnosus* that code for proteins, such as staphylococcal virulence factors, that indicate its pathogenic ancestor [8]. Four of the toxin genes in pathogenic staphylococci that are directly associated with virulence are present in *Staphylococcus carnosus* [9].

When blood cultures are negative, and there is high clinical and radiological suspicion for discitis osteomyelitis, tissue sampling may be needed to identify the causative pathogen to guide antimicrobial therapy [10,11]. A meta-analysis in 2017 showed a significantly high yield for culture results from open biopsy up to 91% compared to 48% yield from image-guided percutaneous biopsy [12]. However, percutaneous tissue sampling is still preferred because of lower complication rates and shorter hospital stays [13,14]. The Infectious Disease Society of America (IDSA) recommends holding antimicrobial therapy for one-two weeks before attempting a biopsy [10]. The yield for percutaneous biopsy could remain low from prior antimicrobial exposure or if the correct site could not be biopsied, and the IDSA recommends repeating the biopsy if the initial biopsy is non-diagnostic and there are no other culture data to guide management [10]. Histopathological evaluation may be of help as imaging findings of discitis osteomyelitis could overlap with other noninfectious causes. However, the criteria for histopathological diagnosis are not clearly defined, and interobserver variability amongst pathologists is high [15,16]. In our patient, a biopsy was performed twice as the initial blood cultures were negative; however, there were not enough specimens available for histopathological analysis. PCR targeting 16S rRNA has been increasingly identified as a valuable tool in addition to cultures specifically for pathogens that are difficult to isolate in culture media; however, the evidence is lacking for discitis osteomyelitis [17,18]. In addition, the availability of molecular testing, transportation of specimens to reference laboratories, and interpretation of the results, meaning differentiating true vs contaminant results, limits its use.

There is no data to support whether *Staphylococcus carnosus* could be misidentified as another coagulase-negative Staphylococcus species. However, there is evidence that *Staphylococcus hominis* being is incorrectly identified based on biochemical and automated identification systems [19]. In our case, when the patient presented for the second time, the blood cultures returned positive in two out of four bottles for *Staphylococcus hominis*, but on the third presentation, all the blood culture bottles were positive for *Staphylococcus carnosus*, which was identified when the specimen was sent to a reference laboratory (ARUP Laboratories, part of the University of Utah Department of Pathology). More studies are needed in this respect, and to determine if species level identification affects treatment outcomes.

The IDSA recommends that a six-week therapy for antibiotics is adequate for most patients with nonspecific spondylodiscitis. For oxacillin-susceptible staphylococci, first-line treatment includes cefazolin or ceftriaxone, and alternative treatment includes vancomycin or daptomycin [20]. In our case, the patient was appropriately treated with a six-week course of IV daptomycin and IV ceftriaxone, with improvement in inflammatory markers such as ESR and CRP, and improvement in symptoms. However, he still had a relapse of infection four months later and required repeated treatment with another course of IV antibiotics therapy. Due to the rapid relapse of infection, we chose to continue oral antibiotics after completion of IV antibiotics to prevent future relapses, keeping in mind the potential harmful effects of prolonged courses of antibiotic therapy, such as *Clostridioides difficile* infection and multidrug-resistant organism infections. Shared decision-making was performed with the patient, who opted to continue antibiotic therapy for symptom relief and to prevent recurrent hospitalization. More studies are needed in this respect to identify the appropriate course of antibiotics, and if there is conclusive evidence to continue oral antibiotics despite improvement in symptoms and inflammatory markers, if the patient continues to show radiological evidence of infection.

## Conclusions

Coagulase-negative Staphylococcus species such as *Staphylococcus carnosus* are uncommon causes of spinal discitis and osteomyelitis. However, this case highlights the importance of considering these factors when managing patients presenting with back pain. Timely initiation of antibiotics after a thorough workup prevents complications and reduces patient morbidity.
